# Clinical validity of second-generation tau PET tracers as biomarkers for Alzheimer’s disease in the context of a structured 5-phase development framework

**DOI:** 10.1007/s00259-020-05156-4

**Published:** 2021-02-16

**Authors:** Gérard N Bischof, Alessandra Dodich, Marina Boccardi, Thilo van Eimeren, Cristina Festari, Henryk Barthel, Oskar Hansson, Agneta Nordberg, Rik Ossenkoppele, Osama Sabri, B Frisoni G Giovanni, Valentina Garibotto, Alexander Drzezga

**Affiliations:** 1 Department of Nuclear Medicine, University Hospital Cologne, Cologne, Germany; 2 NIMTlab, Neuroimaging and Innovative Molecular Tracers Laboratory, University of Geneva, Geneva, Switzerland; 3 Center for Neurocognitive Rehabilitation (CeRiN), CIMeC, University of Trento, Trento, Italy; 4 German Center for Neurodegenerative Disorders (DZNE), Rostock/Greifswald, Rostock, Germany; 5 German Center for Neurodegenerative Disorders (DZNE), Bonn/Cologne, Germany; 6 LANE – Laboratory of Alzheimer’s Neuroimaging and Epidemiology, IRCCS Istituto Centro San Giovanni di Dio Fatebenefratelli, Brescia, Italy; 7 Department of Nuclear Medicine, University of Leipzig, Leipzig, Germany; 8 Memory Clinic, Skåne University Hopsital, Malmö, Sweden; 9 Clinical Memory Research Unit, Department of Clinical Sciences Malmö, Lund University, Lund, Sweden; 10 Division of Clinical Geriatrics, Center for Alzheimer Research, Department of Neurobiology, Care Sciences and Society, Karolinska Institutet, Stockholm, Sweden; 11 Alzheimer Center Amsterdam, Department of Neurology, Amsterdam Neuroscience, Vrije Universiteit Amsterdam, Amsterdam UMC, Amsterdam, Netherlands; 12 Memory Center - Department of Rehabilitation and Geriatrics, Geneva University Hospitals, Geneva, Switzerland; 13 Nuclear Medicine and Molecular Imaging Division, Diagnostic Department, Geneva University Hospitals, Genève, Switzerland; 14 Molecular Organization of the Brain, Institute for Neuroscience and Medicine (INM-2), Jülich, Germany

**Keywords:** Alzheimer’s disease, Strategic roadmap, Biomarker-based diagnosis, Second-generation tau PET tracers

## Abstract

**Purpose:**

In 2017, the Geneva Alzheimer’s disease (AD) strategic biomarker roadmap initiative proposed a framework of the systematic validation AD biomarkers to harmonize and accelerate their development and implementation in clinical practice. Here, we use this framework to examine the translatability of the second-generation tau PET tracers into the clinical context.

**Methods:**

All available literature was systematically searched based on a set of search terms that related independently to analytic validity (phases 1–2), clinical validity (phase 3–4), and clinical utility (phase 5). The progress on each of the phases was determined based on scientific criteria applied for each phase and coded as fully, partially, preliminary achieved or not achieved at all.

**Results:**

The validation of the second-generation tau PET tracers has successfully passed the analytical phase 1 of the strategic biomarker roadmap. Assay definition studies showed evidence on the superiority over first-generation tau PET tracers in terms of off-target binding. Studies have partially achieved the primary aim of the analytical validity stage (phase 2), and preliminary evidence has been provided for the assessment of covariates on PET signal retention. Studies investigating of the clinical validity in phases 3, 4, and 5 are still underway.

**Conclusion:**

The current literature provides overall preliminary evidence on the establishment of the second-generation tau PET tracers into the clinical context, thereby successfully addressing some methodological issues from the tau PET tracer of the first generation. Nevertheless, bigger cohort studies, longitudinal follow-up, and examination of diverse disease population are still needed to gauge their clinical validity.

## Introduction

The recently proposed criteria for the in vivo diagnosis of Alzheimer’s disease (AD) developed by an International Working Group (IWG) [1] and the National Institute of Aging-Alzheimer Association (NIA-AA) task force [2] underline the importance of biomarker assessment in increasing the accuracy of AD diagnosis at predementia and dementia stages. More recently, a “research framework” for observational and interventional research has been added [3]. This framework is based on the AT(N) model, which defines AD as a biological construct [3] and describes subjects on the basis of AD-specific biomarkers targeting amyloid (A) and tau (T) as well as measures of neurodegeneration (N). As a consequence of the lack of a formal structure to guide the development of AD biomarkers, effort has recently been made in order to make the validation of biomarkers for AD more systematic. Specifically, a recent initiative set up a strategic research roadmap, based upon a framework for the validation of biomarkers used in oncology [4], with the aim of improving the validation process required to support the adoption of biomarkers in clinical practice [5, 6]. Previous studies with this shared objective assessed this 5-phase framework with other known biomarkers: cerebrospinal fluid (CSF) assessment of amyloid and tau pathology [7], medial temporal atrophy [8], FDG-PET [9] and ^123^I-ioflupane brain single photon emission tomography, ^123^I-MIBG cardiac scintigraphy [10], and episodic memory impairment [11]. The strategic biomarker roadmap methodology has then been revised (Boccardi et al. [12]) to incorporate the most recent theoretical advancements as well as to accommodate biomarkers of tauopathy.

Tau-PET has recently been introduced among the T biomarkers, and despite the promising preliminary results in the last 5 years, its maturity for standard use in clinical practice is still to be defined. Along with the rapid development of molecular imaging in the last decade, a number of specific tracers targeting tau aggregates have been designed. The recent introduction of tau-specific PET ligands allows evaluating the presence of aggregates of hyper-phosphorylated tau in neurofibrillary tangles in vivo, as well as its topographical distribution. The development of tau tracers has intrinsic complexities, due to the target location, the relatively low target expression, and the high concentration of competing off-target binding sites. The first study which claimed to have successfully accomplished tau PET imaging in humans used the tracer ^18^F-FDDNP [13], which however showed both specific and unspecific (e.g., amyloid-ß) bindings. In the following, numerous other tracers have been synthesized with the aim to develop a tau-selective PET tracer, such as [11C]PBB3, fluorine-labeled tracer such as T807 (AV-1451) and T808 and THK-5351, THK-5105, and THK-5117 (see [14, 15] for reviews). New tracers developed with the aim to reduce potential off-target binding as seen during the validation process of the so-called first-generation tau PET (i.e., tracer of the THK-series, or ^18^F-AV-1451) have been termed second-generation tau PET tracers. This review will systematically assess the development of second-generation tau PET tracers (i.e., ^18^F-RO-948, ^18^F-MK-6240, ^18^F-PI-2620, ^18^F-JNJ-311, ^18^F-GTP1) as biomarkers of AD, by evaluating the achievement of the validation aims defined by the strategic biomarker roadmap (2017; 2020).

## Methods

### Target

The target population is represented by patients with mild cognitive impairment (MCI) and dementia due to AD. Only patients with sporadic (and not familial) AD were considered. AD neuropathology or development of incidental AD dementia (see AD dementia) confirmed via in vivo biomarker of beta-amyloid pathology in cross-sectional studies or with follow-up studies has been considered as the diagnostic reference standard (see Boccardi et al. [12]).

For the aims of the review, only the group of second-generation tau PET tracers was taken into consideration. The evidence for first-generation tau tracers including ^18^F-AV-1451 is reported elsewhere (Wolters et al. [16], Chiotis et al. [17]).

### Glossary

#### Alzheimer’s disease

This term refers to the presence of AD pathology, characterized by extracellular amyloid-β plaques and aggregates of hyper-phosphorylated tau in neurofibrillary tangles, presumably leading to a specific pattern of neurodegeneration (mediotemporal and temporoparietal regions). As such, the term does not indicate the clinical expression or the severity of the disease.

#### AD dementia

Dementia, i.e., acquired and progressive cognitive decline, is associated to a loss of functional autonomy, as defined by the National Institute of Neurological and Communicative Disorders and Stroke and the Alzheimer’s Disease and Related Disorders Association (NINCDS-ADRDA) criteria [18]. Due to the imperfect accuracy of purely clinical criteria, not all clinically defined AD dementia cases have AD pathology.

#### Mild cognitive impairment

This term describes a population with objective cognitive impairment but no functional disability. As it is solely defined based on clinical testing, it includes cases that will progress to AD dementia (about 50%) or non-AD dementia (about 10–15%; [19–21]), or other causes of cognitive impairment such as severe depression (about 35–40%). MCI cases tested positive for AD biomarkers are defined as prodromal AD in the clinical criteria by Dubois et al. (2014) [1].

#### Non-AD neurodegenerative disease

This term defines a group of neurodegenerative disorders often considered for differential diagnosis. The large pathological spectrum includes hippocampal sclerosis, frontotemporal dementia (FTD), Lewy body dementia (LBD), progressive supranuclear palsy (PSP), and corticobasal degeneration (CBD).

### Conceptual framework

The guiding principle of the present framework was based on the phases delineated for biomarker development in oncology by Pepe et al. (2001), with specific adaptations to reflect the screening and diagnosis process for AD (Boccardi et al. [12]). The present review assessed the maturity of the second-generation tau PET tracers with regard to each of the following phases and is detailed below:

#### Phase 1

Phase 1 studies are preclinical exploratory studies aimed at identifying the rational of the biomarker based on pathology findings.

#### Phase 2

This phase includes studies aiming to define the ability of the biomarker to distinguish AD dementia patients from controls. These studies focus on the definition of clinical assay for reliable discrimination, and on the assessment of the possible factors that may influence the threshold for biomarker positivity, such as age, gender, apolipoprotein ε4 (ApoE4) allele carrier status, amyloid positivity, educational level, and comorbidities.

#### Phase 3

Phase 3 evaluates the capacity of the biomarker in detecting the disease in its early phase, in this framework defined by MCI. This includes prospective longitudinal repository studies aimed to define criteria for biomarker positivity, as well as to compare the usefulness of the biomarker with that of the other biomarkers, and their combination in the definition of disease positivity.

#### Phase 4

Studies from this phase aim to estimate the accuracy and usefulness of the biomarker-based diagnosis in representative patients. They consist of prospective diagnostic studies in MCI subjects who may undergo treatment following the biomarker-based diagnosis. They assess the beneficial effect of the biomarker-based detection in terms of early diagnosis, feasibility, compliance, and provide preliminary evidence about costs.

#### Phase 5

Phase 5 studies aim to quantify the impact of the biomarker-based diagnosis on costs and clinically meaningful outcomes (change of management, mortality, morbidity, and quality of life).

### Evidence evaluation

For all phases, evidence was searched in the literature to evaluate whether each aim was considered as achieved, fully achieved, partly achieved, presenting preliminary evidence, or not achieved, as defined in the following list.

1. Fully achieved: Scientific evidence is available and replicated in adequately powered samples in studies without major methodological faults.
2. Partly achieved: Scientific evidence is available but not yet sufficiently replicated, or samples are not adequately powered, or other significant methodological limitations can be found in the available literature.
3. Preliminary evidence: Only preliminary evidence is available.
4. Not achieved: No evidence was found at all, and no studies are known to be ongoing at the time of the writing of the present review.
5. Unsuccessful: Available scientific evidence shows a failure for the biomarker in achieving the aim. Findings in the subsequent roadmap phases should be interpreted with caution.

The fulfillment of each validation step from phase 1 to phase 5 has been assessed consistently with the 2017 Biomarker Roadmap [6]. However, in this initiative, we have performed a data extraction that summarizes the available data, thus allowing the reader to make its own appraisal of aim compliance and provide a sounder evidence assessment. To that end, for each primary and secondary aim of each study, we have extracted data consistent with formal evidence assessment as previously described [26] . Tables with data extraction are accessible online (https://drive.switch.ch/index.php/s/4reUTSuqNZHyIC8​).

### Papers search and selection

The PubMED database was used to search for relevant literature. The search was conducted on October 10 of 2019 and repeated on May 15 of 2020 by author GNB and replicated on 17 September 2020 by CF.

Keywords used to identify research articles utilizing second-generation tau PET tracers are detailed in the online material (accessible via this link: https://drive.switch.ch/index.php/s/4reUTSuqNZHyIC8).

Abstracts and titles of all relevant studies were screened, and articles from other sources, such as references from selected papers or personal knowledge, were added. We report the number for screened, excluded, and finally included articles according to PRISMA guidelines.

## Results

### Current clinical validity of tau-PET imaging

#### Phase 1. Preclinical exploratory studies

##### Primary aim: To identify and prioritize leads for potentially useful biomarkers

Significant off-target binding of first-generation tau PET tracers to monoamine oxidase B (MAO-B) has been shown in basal ganglia and thalamus for all tracers of the THK series, ^11^C-PBB-3, and ^18^F-AV-1451 [22, 23]. Additionally, neuromelanin binding in the substania nigra and binding to biondi ring tangles in the choroid plexus was shown for ^18^F-AV-1451 [24]. Thus, a primary goal for the studies aiming to identify a potential lead for the second-generation tau PET tracers was screening potential candidates against off-target binding while showing high affinity to both 3R and 4R tau deposits. Following the examination of multiple probes, the further development of ^18^F-RO-948 finally showed significant binding to 3R-4R tau isoforms in human brain tissue of AD patients compared to healthy controls, preferable kinetics, and significant brain uptake in a baboon model [25]. Importantly, negligible levels of MAO-B binding [26] and significantly less off-target binding in the choroid plexus compared to ^18^F-AV-1451 could be established [27].

In an elegant design screening for potential tau ligands to significantly reduce binding to both MAO-B and monoamine oxidase A (MAO-A), ^18^F-7, later coined ^18^F-PI-2620, was discovered [26]. Displacement assays with the selective, reversible MAO-A binder fluorethylharmine and the selective, reversible MAO-B deprenyl binder showed non-measurable affinity of 18F-PI2620 towards MAO-A and MAO-B, excellent binding to tau pathology in AD brain tissue of different Braak stages, and favorable kinetics in rhesus monkey models [26].

In the preclinical characterization study for ^18^F-MK-6240, binding to phosphorylated tau in human AD brain tissue could be established [28]. While self-block studies in the rhesus monkey with unlabeled MK6240 did not alter the volume distribution (VT) of ^18^F-MK-6240 in cortex, midbrain, striatum, or thalamus, unlabeled AV1451 showed a marked reduction in VT between baseline and self-block indicative of the known off-target binding [28]. Additionally, ^18^F-MK-6240 does not seem to bind significantly to tau aggregates in non-AD tauopathies, such as progressive supranuclear palsy (PSP), corticobasal degeneration (CBD), or FTD [29].

The Genentech Tau Probe 1 ^18^F-GTP1 provided similar characteristics of no measurable binding to MAO-B in AD tissue, with high affinity to tau pathology in human brain tissue of several AD patients [30].

Finally, in a competitive binding assay, ^18^F-JNJ-311 was showing highly displaceable binding to tau-rich regions particularly in AD, while no specific binding in PSP or CBD was found [31].

In sum, advances in the development of tau PET tracers of the second-generation have overcome significant shortcomings with respect to the known off-target binding sites [32] of the first tau-PET tracer generation. Importantly, these developments allow a better assessment of the utility of these tracers to detect secondary and/or primary tauopathies. Although not all potential off-target binding sites for the second-generation tau PET tracers have been examined, it appears, however, that skull and meningeal uptake is more prevalent across this group of tracers [27, 33]. For some second-generation tau PET tracers, this specific pattern of off-target binding does not influence the diagnostic accuracy, when sampling tau pathology from different Braak regions to dissociate AD from healthy controls as it was exemplary shown for ^18^F-RO-948 [27]. However, studies examining ^18^F-MK-6240 reported that off-target meninges binding may spill into adjacent cortical areas [33].

**Fig. 1:**
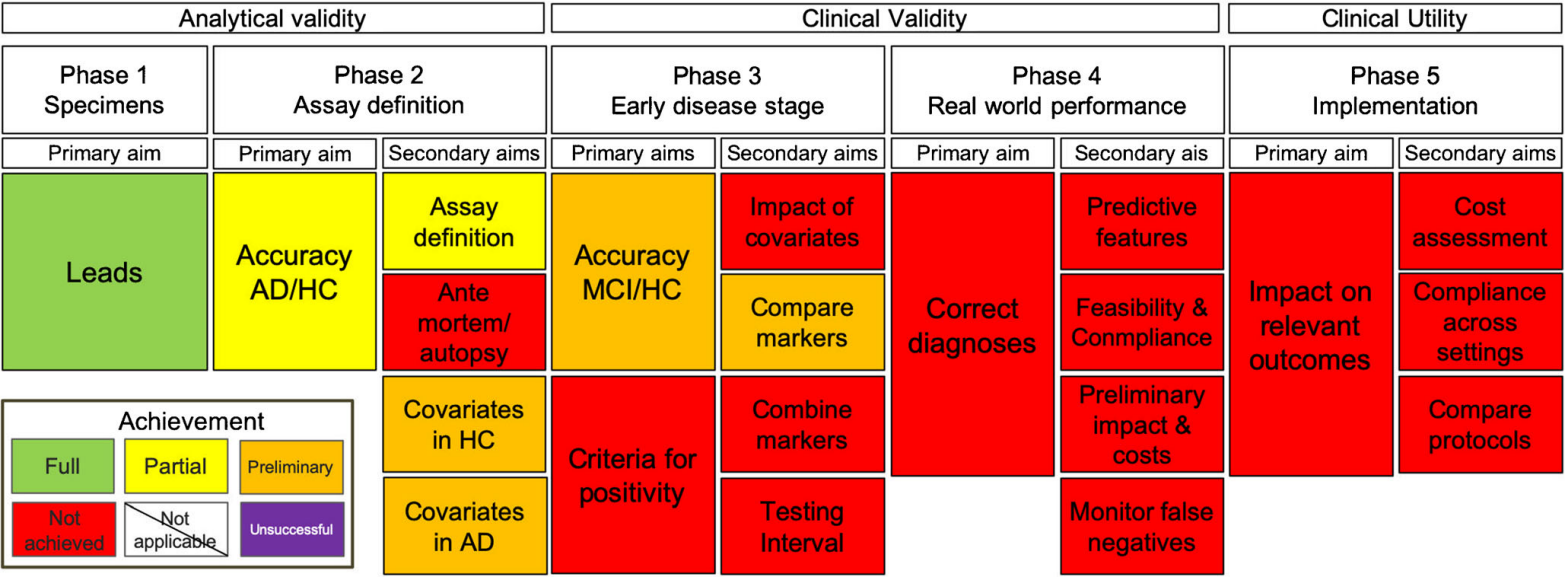
Current state of second-generation tau PET tracers in the context of the strategic biomarker roadmap (adapted from Frisoni et al., 2017)

We conclude that the primary aim of phase 1 has been fully achieved (*see* Fig. 1) as enough scientific evidence has been produced to identify potential leads for the second-generation tau PET tracers.

#### Phase 2. Clinical assay development for Alzheimer’s disease pathology

##### Phase 2. Primary aim: To estimate true-positive and false-positive rates, or receiving operating characteristics curves (ROC) for the assay and to identify the discrimination accuracy between subjects with and without the disease

To date, only data for ^18^F-RO-948 are published [34], as multicenter studies with sufficient sample sizes for the majority of reviewed compounds are underway. This study included healthy controls (*N* = 257), patients diagnosed with MCI (*N* = 154) and AD (*N* = 100), and a group of non-AD patients (*N* = 100) including patients with behavioral variant of FTD and semantic variant of primary progressive aphasia, dementia with Lewy bodies, multiple system atrophy, vascular dementia, and patients on the Parkinson spectrum with and without dementia and PSP. 18F-RO-948 signal retention was sampled from regions reflecting Braak stages I–II, Braak stages III–IV, Braak stages I–IV, and Braak stages V–VI. The area under the curves (AUCs) for discriminating AD (*N* = 100) from healthy controls (*N* = 257) for the summary measure of regions compromising Braak stages I–IV equaled 0.98 (0.96–0.99), whereas the AUC for AD vs non-AD patients was 0.97 (0.95–0.99). Of note, this study did not provide pathological confirmation for the observed pattern of ^18^F-RO-948 which is the desired reference standard for phase 2 on clinical validity. Nevertheless, this is the first and biggest study to date, providing accuracy estimates for the discrimination of individuals with and without the disease.

##### Phase 2. Secondary aim 1: To optimize procedures for performing the assay and to assess its reproducibility within/between laboratories

Several groups have examined in vivo kinetics and different quantification strategies for ^18^F-MK-6240 [35–38]. They all included patients with a diagnosis of probable AD, patients with MCI, and healthy controls who underwent dynamic PET imaging between 120 and 180 min. Arterial blood sampling to further evaluate kinetic modeling was provided only for a subset of patients [35, 37, 38]. Overall, the majority of studies concluded that ^18^F-MK-6240 demonstrated high affinity for neurofibrillary tangles in MCI and AD subjects with a dynamic and wide range. Static standardized uptake value ratios (SUVRs) corresponded well with kinetic modeling-derived readouts. Interestingly, different static time windows have been suggested for SUVR-based methods varying between 90 to 110 min [35] and 70 to 90 min [36, 37]. Additionally, and as previously observed with the set of first-generation tau PET tracers, ^18^F-MK-6240 SUVR measurements did not reach equilibrium in high binding regions [38], which could influence quantitative interpretation especially in longitudinal studies or treatment trials. Only one appropriate test-retest design has been performed with ^18^F-MK-6240, where both arterial blood sampling and dynamic scanning were repeated between 3 days and 3 weeks following the baseline scan [39]. Study results suggest that SUVR measurements of 90–120 min were least affected by test-retest variability (~ 6%), which is somewhat higher than variability values reported for SUVR measurement of ^18^F-AV1451 ((~ 3%), for details *see* Wolters et al. [16]).

Quantification strategies have been explored for ^18^F-PI-2620 with dynamic scanning protocols up to 180 min and arterial blood sampling in AD patients and healthy controls, but not patients with MCI [40, 41]. Earlier time windows (i.e., p.i. 40–75 min) for static imaging have been suggested for ^18^F-PI-2620 compared to other second-generation tau PET tracers. Additionally, secular equilibrium around 40 min was observed even in tau-rich regions suggesting that quantitative interpretation may overall be easier with ^18^F-PI-2620. Only for a small proportion of subjects (*N* = 3 HC; *N* = 4 AD), test-retest variability measures were analyzed and 3.5% (30–60 min) and 4.3% (45–75 min) variability for the respective time windows was observed [41].

Only one study reported on quantification approaches for ^18^F-RO-948 using a dynamic scanning protocol up to 200 min and arterial blood sampling in AD, MCI, and healthy control subjects [42]. Homogeneous radioactivity distribution was observed 60–90 min p.i., in healthy control subjects, whereas AD cases showed a heterogenous pattern of tracer retention. Similar to ^18^F-MK-6240, SUVR equilibrium in some AD cases was not reached until 150 min. The majority of groups has decided on a 70–90 min p.i. time window for static ^18^F-RO-948 scanning protocols [27, 34].

Detailed quantification approaches have been provided for almost all tau PET tracers of the second-generation, except ^18^F-JNJ-311 and ^18^F-GTP1. Due to the partial lack of appropriate test-retest designs to evaluate reproducibility and variability of tracer retention, this aim has only been preliminary achieved (*see* Fig. 1).

##### Phase 2. Secondary aim 2: To determine the relationship between biomarker measurements made on brain tissue and the biomarker measurements made on the non-invasive clinical specimen

End-of-life studies examining the correspondence of antemortem tracer retention and postmortem tau pathology have yet to be provided for any of the second-generation tau tracers. Investigations from the first-generation tau PET tracer ^18^F-AV1451 indicate an increasing complexity when detecting earlier than later Braak tau stages [43, 44] and should be considered in the respective study designs. As evidence is potentially underway for the second-generation tau PET tracers, this aim has not yet been achieved (*see* Fig. 1).

##### Phase 2. Secondary aim 3: To assess factors (e.g., sex, age, etc.), associated with biomarker status or level in control subjects

Assessments on possible sex differences in the uptake patterns of the second-generation tau PET tracers have been provided for ^18^F-MK-6240 only [45]. The analysis was performed in a subset of cognitively healthy subjects comparing retention patterns between 50 Hispanic females and 25 Hispanic males. The authors reported that tau pathology in middle/inferior gyri was significantly higher in females than males in addition to higher global amyloid burden for females compared to males. It is noteworthy that the study design included adults with a mean age of 64.0 (SD = 3.43) who were all from the Hispanic community in the USA as the authors focused on investigating the prevalence of AD pathology in younger Hispanic age groups.

Associations of retrospective cognitive decline and elevation of retention of ^18^F-MK-6240 were performed in a larger sample (*N* = 167) of healthy middle-aged adults (mean age of enrollment = 54 years) with a parental history of AD [46]. Importantly, the authors adopted a biomarker-based definition of AD [6] and categorized all individuals in the sample into amyloid positive/negative and tau positive (as determined as 2 STD above the mean of enthorinal tau of the amyloid negative group)/negative and examined the trajectories on a summary cognitive measure retrospectively. The authors observed that amyloid-positive/tau-positive individuals (*N* = 15) showed the most precipitous cognitive decline compared to the other groups. Importantly, sensitivity analysis either using different thresholds for tau positivity or examining different tau-relevant regions (e.g., hippocampus, amygdala) underscored the robustness of their main finding. Additionally, age-related correlation of ^18^F-MK-6240 was small and disappeared when controlled for amyloid status [46]. Further evidence provided from the same cohort underscored the importance of amyloid deposition, showing that amyloid chronicity, a measure of exposure to significant amyloid burden over time, was associated with retention of ^18^F-MK-6240 in the entorhinal cortex [47].

In addition to sex, ethnicity, amyloid status, and cognition, other covariates may influence signal retention of tau pathology in cognitively healthy subjects (i.e., education, APOE status). If healthy controls will be used to establish thresholds of positivity for diseased populations, it will be of importance to further understand the synergy of these effects. Complete inferences cannot yet be drawn from the very preliminary evidence produced on this topic (*see* Fig. 1).

##### Phase 2. Secondary aim 4: To assess factors associated with biomarker status or level in cognitively impaired subjects—in particular, disease characteristics such as stage, molecular features and prognosis

Cross-sectional evidence on the association of 18FGTP1 retention and cognitive scores was recently shown for a mixed sample of cognitively normal adults (*N* = 10), prodromal (*N* = 27), mild (*N* = 19), and moderate (*N* = 15) AD cases [48]. Interestingly, signal retention of global measures of ^18^F-GTP1 (whole cortical gray matter) was associated with global cognitive measures such as MMSE, CDR-SB, ADAS-COG13, and RBANS, but not measures of beta-amyloid burden. Additionally, regional sensitivity of tau burden sampled from temporal regions was shown by negative correlations with these measures and delayed memory performance (i.e., story recall). Further research using this compound in cognitively normal adults (*N* = 11), prodromal (*N* = 15), mild (*N* = 13), and moderate (*N* = 10) AD cases showed a significant association of CSF measure, including p-tau, t-tau, and CSF tau368 fragment, and GTP-1 retention in a temporal region of interest [49]. Importantly, these associations remained significant after accounting for age differences but were somewhat reduced. As more studies are underway, we conclude that preliminary evidence on the possible variables influencing or associated with signal retention at various clinical stages has been provided (*see* Fig. 1).

#### Phase 3. Retrospective/prospective longitudinal repository studies

##### Phase 3. Primary aim 1: To evaluate the capacity of the biomarker to predict conversion to AD dementia in the prodromal stage (MCI) as a function of time

To date, only one study [34] investigated the ability of a second-generation tau PET tracer to detect significant tau pathology early in the disease cascade. Specifically, signal retention of ^18^F-RO-948 showed an AUC = 0.80 (0.75–0.85), when compared in patients diagnosed with MCI and positive on CSF-markers of beta-amyloid (*N* = 96) to healthy controls (*N* = 257), whereas the AUC for the comparisons with the non-AD patient group was somewhat lower (AUC = 0.73 (0.66–0.80)). It is noteworthy that sensitivity measures in all comparisons with amyloid-positive MCI patients varied substantially depending from which Braak stage regions signal retention was sampled from (Range: 13.0–47.0). In conclusion, preliminary evidence has been provided for 18F-RO-948 and its ability to discriminate between healthy and diseased population. To date, there are no longitudinal studies available examining the ability of second-generation tau PET tracers to predict the conversion from MCI to AD dementia.

##### Phase 3. Primary aim 2: Define criteria for a positive diagnostic test for MCI due to AD, in preparation of phase 4

A desired goal in the current research field that assesses in vivo tau pathology using PET biomarker is the definition of an accurate threshold for tau positivity. The goal, however, is not trivial as in vivo measures of tau pathology appear to be much more vulnerable to various covariates, and these relationships are not always linear [50], and they change as the disease progresses. Although different initial approaches have been suggested for both first- and second-generation tau PET tracers such as sampling from multiple regions constituting different Braak stages [34], or sampling from early tau-rich regions such as temporal regions [51] in comparison to low tracer retention from a young healthy control cohort, such efforts have yet to mature for the group of second-generation tau PET tracers (*see* Fig. 1).

##### Phase 3. Secondary aim 1: To explore the impact of relevant covariates on the biomarker discrimination abilities before the clinical diagnosis

This aim has not been successfully achieved for any of the current available tau PET tracers if considering evidence from the first- or the second-generation compounds. However, the currently available data [34] on the lower discrimination performance of ^18^F-RO-948 between positive MCI patients and AD or non-AD patients (compared to high discrimination between HC vs. AD) suggests that that the influence of covariates needs to be further explored.

##### Phase 3. Secondary aim 2: To compare the different biomarkers in order to select those that seem most promising

There are currently two studies [27, 34] that directly or indirectly compared a second-generation tau PET tracer (i.e., ^18^F-RO-948) to other biomarkers assessing the same pathology (e.g., CSF measures of phosphorylated tau (p-tau) or to a first-generation tau PET tracer ^18^F-AV1451). Whereas regional summary measures of ^18^F-RO-948 sampled from regions associated with Braak stages outperformed measures of p-tau 181 in distinguishing AD, healthy controls and non-AD patients, ratio measures of both CSF Abeta 40/Abeta 42 and CSF Abeta 42/p-tau 181 showed better discrimination performance for the comparison of MCI (amyloid positives) vs. non-AD disorders. Head-to-Head comparison between ^18^F-RO-948 and ^18^F-AV-1451 was primarily designed to assess differences in off-target binding of the two compounds. The authors showed that the amount of off-target binding observed for ^18^F-RO-948 was significantly reduced in choroid plexus, basal ganglia, and thalamus. ^18^F-RO-948, however, showed significantly more uptake in skull and meninges than ^18^F-AV-1451, particularly in cases where there is little tracer retention in cortex. Comparing diagnostic accuracy by removing individuals with elevated off-target uptake in skull and meninges showed that discrimination performance between healthy controls and AD patients was not significantly affected. It is important to note here that the sample size (AD *N* = 18, MCI-AD *N* = 3, and controls *N* = 4) was not primarily designed to assess discrimination performance between diseased and healthy population on a larger scale. Together, biomarker comparison studies are still scarce, and it is feasible to assume that different types of biomarker assessments will have different accuracy in the prognostic and diagnostic process.

##### Phase 3. Secondary aim 3: To develop algorithms for the biomarker-based diagnosis of MCI in preparation of phase 4

As there are no longitudinal studies at the moment for any tau PET tracer, the development of algorithms that allows an accurate prediction of the cognitive deterioration of patients with MCI is still in progress. In the context of the assessed framework, this goal has not been achieved.

##### Phase 3. Secondary aim 4: To determine an interval able to detect a meaningful change of biomarker status or level in progressing MCI

While longitudinal studies for the first-generation tau PET tracer have been examined up to f4 year follow-up in MCI patients [52, 53], longitudinal studies for the second-generation tau PET tracers including prodromal disease stages are still underway.

## Discussion

Here, we adopted a strategic biomarker roadmap originally proposed for the biomarker development in oncology [4] to assess the maturity of second-generation tau PET tracers to diagnose AD in view of their translation to the clinical context. The guiding principles allowed to state which stages of the roadmap have been successfully achieved, and the results allow for clear recommendation where more research is needed in order to move the second-generation tau PET tracers into the clinical context.

Whereas the progress of the group of tracers as a whole is by far less advanced compared to the first-generation tau PET tracer ^18^F-AV1451, the major goal of developing new molecular ligands to reduce off-target binding has been achieved by the majority of the presented compounds here. In the following, we will elaborate on the here identified areas in need of urgent programs of research and which methodological challenges should be considered with high priority to move forward with the translation of the second-generation tau PET tracers into the clinical context.

The primary aim of phase 1 of the roadmap has been successfully achieved. Importantly, as the search for novel molecules was guided by lower affinity to other enzyme targets such as MAO-A and MAO-B, the published evidence validates the successful effort in significantly reducing off-target binding properties of the second-generation tau PET tracers [26, 28]. These achievements should, in principle, translate into higher sensitivities to detect early stages of both secondary and primary tauopathies, as there is less or virtually no correspondence between off-target and target signal.

For example, currently ^18^F-MK-6240 has been shown to present strong affinity to 3R/4R tauopathy in AD, whereas affinity to tau filaments prevalent in FTD, CTE, or PSP was low [29]. On the other hand, in vivo studies in both 3R/4R tauopathy in AD [41] and 4R tauopathies in patients with PSP [54] using ^18^F-PI-2620 showed that this ligand can be successfully utilized to discriminate both patient populations (AD and PSP) from healthy controls.

The gold standard for in vivo tracer validation is studies comparing antemortem tracer retention with postmortem histopathological quantification. During the development of the first-generation tau PET tracers [55], reliable confirmation from gold standard studies was widely missing, despite their application into the clinical and research context.

This also translates to the group of second-generation tracers alike, where validation from postmortem studies for the correspondence of tangle pathology and antemortem measures of tracer retention are still being awaited. Specifically, programs of research for the quantification of tau positivity in early and advanced cases of AD need to receive immediate attention as resulting evidence would potentially guide proper scanning protocols, the validation of visual reading guidelines, and improved estimation of tau load as a function of disease. This gold standard has recently been provided for ^18^F-AV1451 [43], which addressed some challenges associated with the translation of neuropathological staging to measurement of continuous in vivo tau pathology into an accurate visual reading protocol. As noted already, we assume that these challenges will be adequately considered in the future gold standard studies of the group of second-generation tau PET tracers.

As proposed in the updated guidelines of the strategic biomarker roadmap (Boccardi et al. [12]) and discussed in the workshop held in Geneva, November 11–12, 2019, the definition of an appropriate standard for diagnostic biomarkers is a critical step in the validation process. Pathology studies are necessary to demonstrate analytical validity. Since the access to this gold standard is a main hurdle in the research on tau PET ligands, other reference standards have been admitted. These are, for example, studies assessing longitudinal progression of tau PET biomarkers and subsequent cognitive decline. Nonetheless, the analytical validity primarily rests on pathological confirmation which is regarded as a major limitation for the group of second-generation tau PET tracers and identified as the most urgent research priority in the field. The evidence reviewed here on the second-generation tau PET tracers do suggest (a) that signal retention of the tau PET compounds in combination with amyloid positivity, particularly in asymptomatic subjects, conveys a significant risk for precipitous cognitive decline and (b) elevated signal retention tracks cognitive deterioration in mild und advanced symptomatic cases. Therefore, despite the lack of gold standard studies, in vivo measurements with the second-generation tau PET tracers provided some convincing evidence of these ligands to serve as a valid diagnostic biomarker in the future.

The overall lack of longitudinal data on the second-generation tau PET tracers complicates the interpretation of individual prediction of elevated tau pathology and subsequent cognitive decline or conversion to prodromal or more advanced disease stages. Initial longitudinal evidence on the first-generation tau PET tracers is promising if they can be translated similarly for the tau PET tracer of the second-generation [50, 52, 56] . Nevertheless, the missing evidence prevents us from interpreting the majority of aims formulated in the strategic biomarker roadmap for phase 3. Additionally, similar studies as presented for ^18^F-RO-948 [34] are needed for other second-generation tau PET tracers and hopefully will provide part of the missing evidence highlighted here.

Several methodological challenges complicate the translatability of the second-generation tau PET tracers into the clinical context: One challenge is the comparability among the second-generation tau PET tracer. Specifically, as head-to-head studies are needed, radiation safety and practical issues may prevent collecting enough data points that ensures an appropriate comparison among the group of compounds. Possibly a careful matching between datasets by clinical severity and multiple demographic variables would be an alternative approach that allows a fair comparison. Such an approach would be particularly suitable for tracer groups that have a high molecular structural resemblance such as ^18^F-RO-948, ^18^F-PI-2620, and ^18^F-GTP1.

The interpretation of longitudinal studies/therapeutic trails to test disease-modifying interventions is challenged by the fact that some AD cases with tau rich regions do not show tracer equilibrium within a comparable time frame [38, 42]. Therefore, SUVR measurements will be inaccurate in such cases, and potential disease progression or treatment efficacy may be masked by this inaccuracy. It is important to adhere to strict timing regimens or employ dynamic scanning protocols as it has been suggested for ^11^C-PIB [57] in studies with longitudinal designs or therapeutic trails [58].

## Conclusion

Here we examined the research evidence on all known second-generation tau PET tracers within the context of the updated strategic biomarker roadmap. Whereas the specimen identification (phase 1) was considered successfully achieved, assay definition (phase2) was only addressed by a fraction of the available research evidence. Consequently, assessments that assist in interpreting the clinical validity (phase 3: early disease stage) was mostly not achieved. Several clinical trials have been registered for various compounds of the second-generation tau PET tracers (i.e., ^18^F-GTP1, ^18^F-MK-6240, ^18^F-PI-2620; Accessed https://clinicaltrials.gov/ 07/06/2020), and fast progress on the strategic biomarker roadmaps is expected (phases 4 and 5). Additionally, our work has identified several areas of much needed research that would assist in advancing the translatability of the second-generation tau PET tracers into the clinical context.

## References

[1] Dubois B, Feldman HH, Jacova C, Hampel H, Molinuevo JL, Blennow K (2014). Advancing research diagnostic criteria for Alzheimer’s disease: the IWG-2 criteria. Lancet Neurol.

[2] Jack CR, Albert MS, Knopman DS, McKhann GM, Sperling RA, Carrillo MC (2011). Introduction to the recommendations from the National Institute on Aging-Alzheimer’s association workgroups on diagnostic guidelines for Alzheimer’s disease. Alzheimers Dement.

[3] Jack CR, Bennett DA, Blennow K, Carrillo MC, Dunn B, Haeberlein SB (2018). NIA-AA research framework: toward a biological definition of Alzheimer’s disease. Alzheimers Dement.

[4] Pepe MS, Etzioni R, Feng Z, Potter JD, Thompson ML, Thornquist M (2001). Phases of biomarker development for early detection of cancer. J Natl Cancer Inst Oxford Academic.

[5] Frisoni GB, Boccardi M, Barkhof F, Blennow K, Cappa S, Chiotis K (2017). Strategic roadmap for an early diagnosis of Alzheimer’s disease based on biomarkers. Lancet Neurol.

[6] Boccardi M, Gallo V, Yasui Y, Vineis P, Padovani A, Mosimann U, et al. The biomarker-based diagnosis of Alzheimer’s disease. 2-lessons from oncology. 152 [Internet]. ELSEVIER; 2017 [cited 2020 Sep 16]; Available from: http://spiral.imperial.ac.uk/handle/10044/1/47894 10.1016/j.neurobiolaging.2017.01.021 28317645

[7] Mattsson N, Lönneborg A, Boccardi M, Blennow K, Hansson O (2017). Geneva task force for the roadmap of Alzheimer’s biomarkers. Clinical validity of cerebrospinal fluid Aβ42, tau, and phospho-tau as biomarkers for Alzheimer’s disease in the context of a structured 5-phase development framework. Neurobiol Aging.

[8] Ten Kate M, Barkhof F, Boccardi M, Visser PJ, Jack CR, Lovblad K-O (2017). Clinical validity of medial temporal atrophy as a biomarker for Alzheimer’s disease in the context of a structured 5-phase development framework. Neurobiol Aging.

[9] Garibotto V, Herholz K, Boccardi M, Picco A, Varrone A, Nordberg A (2017). Clinical validity of brain fluorodeoxyglucose positron emission tomography as a biomarker for Alzheimer’s disease in the context of a structured 5-phase development framework. Neurobiol Aging.

[10] Sonni I, Ratib O, Boccardi M, Picco A, Herholz K, Nobili F (2017). Clinical validity of presynaptic dopaminergic imaging with 123I-ioflupane and noradrenergic imaging with 123I-MIBG in the differential diagnosis between Alzheimer’s disease and dementia with Lewy bodies in the context of a structured 5-phase development framework. Neurobiol Aging.

[11] Cerami C, Dubois B, Boccardi M, Monsch AU, Demonet JF, Cappa SF (2017). Clinical validity of delayed recall tests as a gateway biomarker for Alzheimer’s disease in the context of a structured 5-phase development framework. Neurobiol Aging.

[12] Boccardi M, Dodich A, Albanese E, Gayet-Ageron A, Festari C, Ramusino M, et al. The strategic biomarker roadmap for the validation of Alzheimer’s diagnostic biomarkers: methodological update. EJNMMI. 10.1007/s00259-020-05120-2. 10.1007/s00259-020-05120-2 PMC8175304 33688996

[13] Shoghi-Jadid K, Small GW, Agdeppa ED, Kepe V, Ercoli LM, Siddarth P (2002). Localization of neurofibrillary tangles and beta-amyloid plaques in the brains of living patients with Alzheimer disease. Am J Geriatr Psychiatry.

[14] Leuzy A, Chiotis K, Lemoine L, Gillberg PG, Almkvist O, Rodriguez-Vieitez E, et al. Tau PET imaging in neurodegenerative tauopathies-still a challenge. Mol Psychiatry. 2019;24(8):1112–34. 10.1038/s41380-018-0342-8 10.1038/s41380-018-0342-8 PMC6756230 30635637

[15] Bischof GN, Endepols H, van Eimeren T, Drzezga A (2017). Tau-imaging in neurodegeneration. Methods..

[16] Wolters EE, Dodich A, Boccardi M, et al. Clinical validity of increased cortical uptake of [18F]flortaucipir on PET as a biomarker for Alzheimer’s disease in the context of a structured 5-phase biomarker development framework. Eur J Nucl Med Mol Imaging. 2021. 10.1007/s00259-020-05118-w. 10.1007/s00259-020-05118-w PMC8175307 33547556

[17] Chiotis K, Dodich A, Boccardi M, Festari C, Drzezga A, Hansson O, Ossenkoppele R, Frisoni GB, Garibotto V, Nordberg A. Clinical validity of increased cortical uptake of first-generation Tau PET tracers as a biomarker for Alzheimer’s disease in the context of a structured 5-phase development framework (in preparation for this issue).

[18] McKhann G, Drachman D, Folstein M, Katzman R, Price D, Stadlan EM (1984). Clinical diagnosis of Alzheimer’s disease: report of the NINCDS-ADRDA work group under the auspices of Department of Health and Human Services Task Force on Alzheimer’s disease. Neurology..

[19] Bennett DA, Wilson RS, Schneider JA, Evans DA, Beckett LA, Aggarwal NT (2002). Natural history of mild cognitive impairment in older persons. Neurology..

[20] Jack CR, Lowe VJ, Senjem ML, Weigand SD, Kemp BJ, Shiung MM (2008). 11C PiB and structural MRI provide complementary information in imaging of Alzheimer’s disease and amnestic mild cognitive impairment. Brain..

[21] Rowe CC, Ellis KA, Rimajova M, Bourgeat P, Pike KE, Jones G (2010). Amyloid imaging results from the Australian imaging, biomarkers and lifestyle (AIBL) study of aging. Neurobiol Aging.

[22] Harada R, Ishiki A, Kai H, Sato N, Furukawa K, Furumoto S (2018). Correlations of 18F-THK5351 PET with postmortem burden of tau and astrogliosis in Alzheimer disease. J Nucl Med.

[23] Vermeiren C, Motte P, Viot D, Mairet-Coello G, Courade J-P, Citron M (2018). The tau positron-emission tomography tracer AV-1451 binds with similar affinities to tau fibrils and monoamine oxidases. Mov Disord.

[24] Marquié M, Verwer EE, Meltzer AC, Kim SJW, Agüero C, Gonzalez J (2017). Lessons learned about [F-18]-AV-1451 off-target binding from an autopsy-confirmed Parkinson’s case. Acta Neuropathol Commun.

[25] Gobbi LC, Knust H, Körner M, Honer M, Czech C, Belli S (2017). Identification of three novel radiotracers for imaging aggregated tau in Alzheimer’s disease with positron emission tomography. J Med Chem.

[26] Kroth H, Oden F, Molette J, Schieferstein H, Capotosti F, Mueller A (2019). Discovery and preclinical characterization of 18FPI-2620, a next-generation tau PET tracer for the assessment of tau pathology in Alzheimer’s disease and other tauopathies. Eur J Nucl Med Mol Imaging.

[27] Smith R, Schöll M, Leuzy A, Jögi J, Ohlsson T, Strandberg O (2020). Head-to-head comparison of tau positron emission tomography tracers 18Fflortaucipir and 18FRO948. Eur J Nucl Med Mol Imaging.

[28] Hostetler ED, Walji AM, Zeng Z, Miller P, Bennacef I, Salinas C (2016). Preclinical characterization of 18F-MK-6240, a promising PET tracer for in vivo quantification of human neurofibrillary tangles. J Nucl Med.

[29] Aguero C, Dhaynaut M, Normandin MD, Amaral AC, Guehl NJ, Neelamegam R (2019). Autoradiography validation of novel tau PET tracer [F-18]-MK-6240 on human postmortem brain tissue. Acta Neuropathol Commun.

[30] Sanabria Bohórquez S, Marik J, Ogasawara A, Tinianow JN, Gill HS, Barret O (2019). 18FGTP1 (Genentech tau probe 1), a radioligand for detecting neurofibrillary tangle tau pathology in Alzheimer’s disease. Eur J Nucl Med Mol Imaging.

[31] Declercq L, Rombouts F, Koole M, Fierens K, Mariën J, Langlois X, Andrés JI, Schmidt M, Macdonald G, Moechars D, Vanduffel W, Tousseyn T, Vandenberghe R, Van Laere K, Verbruggen A, Bormans G. Preclinical evaluation of 18F-JNJ64349311, a novel PET tracer for tau imaging. J Nucl Med. 2017;58(6):975–981. 10.2967/jnumed.116.185199. 10.2967/jnumed.116.185199 28232614

[32] Murugan NA, Chiotis K, Rodriguez-Vieitez E, Lemoine L, Ågren H, Nordberg A (2019). Cross-interaction of tau PET tracers with monoamine oxidase B: evidence from in silico modelling and in vivo imaging. Eur J Nucl Med Mol Imaging.

[33] Betthauser TJ, Cody KA, Zammit MD, Murali D, Converse AK, Barnhart TE (2019). In vivo characterization and quantification of neurofibrillary tau PET radioligand 18F-MK-6240 in humans from Alzheimer disease dementia to young controls. J Nucl Med.

[34] Leuzy A, Smith R, Ossenkoppele R, Santillo A, Borroni E, Klein G, et al. Diagnostic performance of RO948 F 18 tau positron emission tomography in the differentiation of alzheimer disease from other neurodegenerative disorders. JAMA Neurol. 2020;77(8):955–65. 10.1001/jamaneurol.2020.0989. 10.1001/jamaneurol.2020.0989 PMC7215644 32391858

[35] Pascoal TA, Shin M, Kang MS, Chamoun M, Chartrand D, Mathotaarachchi S (2018). In vivo quantification of neurofibrillary tangles with 18FMK-6240. Alzheimers Res Ther.

[36] Betthauser TJ, Lao PJ, Murali D, Barnhart TE, Furumoto S, Okamura N (2017). In vivo comparison of tau radioligands 18F-THK-5351 and 18F-THK-5317. J Nucl Med.

[37] Lohith TG, Bennacef I, Vandenberghe R, Vandenbulcke M, Salinas CA, Declercq R (2019). Brain imaging of Alzheimer dementia patients and elderly controls with 18F-MK-6240, a PET tracer targeting neurofibrillary tangles. J Nucl Med.

[38] Guehl NJ, Wooten DW, Yokell DL, Moon S-H, Dhaynaut M, Katz S (2019). Evaluation of pharmacokinetic modeling strategies for in-vivo quantification of tau with the radiotracer 18FMK6240 in human subjects. Eur J Nucl Med Mol Imaging.

[39] Salinas C, Lohith TG, Purohit A, Struyk A, Sur C, Bennacef I, et al. Test-retest characteristic of [18F]MK-6240 quantitative outcomes in cognitively normal adults and subjects with Alzheimer's disease. J Cereb Blood Flow Metab. 2020;40(11):2179–87. 10.1177/0271678X19887781. 10.1177/0271678X19887781 PMC7585918 31711342

[40] Mueller A, Bullich S, Barret O, Madonia J, Berndt M, Papin C (2020). Tau PET imaging with 18F-PI-2620 in patients with Alzheimer disease and healthy controls: a first-in-humans study. J Nucl Med.

[41] Bullich S, Barret O, Constantinescu C, Sandiego C, Mueller A, Berndt M (2020). Evaluation of dosimetry, quantitative methods, and test–retest variability of 18F-PI-2620 PET for the assessment of tau deposits in the human brain. J Nucl Med Society of Nuclear Medicine.

[42] Kuwabara H, Comley RA, Borroni E, Honer M, Kitmiller K, Roberts J (2018). Evaluation of 18F-RO-948 PET for quantitative assessment of tau accumulation in the human brain. J Nucl Med.

[43] Fleisher AS, Pontecorvo MJ, Devous MD, Lu M, Arora AK, Truocchio SP (2020). Positron emission tomography imaging with 18Fflortaucipir and postmortem assessment of Alzheimer disease neuropathologic changes. JAMA Neurol.

[44] Lowe VJ, Lundt ES, Albertson SM, Min H-K, Fang P, Przybelski SA (2020). Tau-positron emission tomography correlates with neuropathology findings. Alzheimers Dement.

[45] Luchsinger JA, Palta P, Rippon B, Soto L, Ceballos F, Pardo M (2020). Sex differences in in vivo Alzheimer’s disease neuropathology in late middle-aged Hispanics. J Alzheimers Dis.

[46] Betthauser TJ, Koscik RL, Jonaitis EM, Allison SL, Cody KA, Erickson CM (2020). Amyloid and tau imaging biomarkers explain cognitive decline from late middle-age. Brain..

[47] Koscik RL, Betthauser TJ, Jonaitis EM, Allison SL, Clark LR, Hermann BP, et al. Amyloid duration is associated with preclinical cognitive decline and tau PET. Alzheimer’s & Dementia : Diagnosis, Assessment & Disease Monitoring [Internet]. Wiley-Blackwell; 2020 [cited 2020 Sep 21];12. Available from: https://www.ncbi.nlm.nih.gov/pmc/articles/PMC7085284/10.1002/dad2.12007 PMC7085284 32211502

[48] Teng E, Ward M, Manser PT, Sanabria-Bohorquez S, Ray RD, Wildsmith KR (2019). Cross-sectional associations between 18FGTP1 tau PET and cognition in Alzheimer’s disease. Neurobiol Aging.

[49] Blennow K, Chen C, Cicognola C, Wildsmith KR, Manser PT, Bohorquez SMS (2020). Cerebrospinal fluid tau fragment correlates with tau PET: a candidate biomarker for tangle pathology. Brain Oxford Academic.

[50] Jack CR, Wiste HJ, Schwarz CG, Lowe VJ, Senjem ML, Vemuri P (2018). Longitudinal tau PET in ageing and Alzheimer’s disease. Brain Oxford Academic.

[51] Harrison TM, La Joie R, Maass A, Baker SL, Swinnerton K, Fenton L (2019). Longitudinal tau accumulation and atrophy in aging and alzheimer disease. Ann Neurol.

[52] Cho H, Choi JY, Lee HS, Lee JH, Ryu YH, Lee MS (2019). Progressive tau accumulation in Alzheimer disease: 2-year follow-up study. J Nucl Med.

[53] Chiotis K, Savitcheva I, Poulakis K, Saint-Aubert L, Wall A, Antoni G, et al. [18F]THK5317 imaging as a tool for predicting prospective cognitive decline in Alzheimer's disease. Mol Psychiatry. 2020. 10.1038/s41380-020-0815-4. 10.1038/s41380-020-0815-4 PMC8758479 32616831

[54] Brendel M, Barthel H, Eimeren T van, Marek K, Beyer L, Song M, et al. Assessment of 18F-PI-2620 as a biomarker in progressive supranuclear palsy. JAMA Neurol [Internet]. 2020 [cited 2020 Sep 22]; Available from: https://jamanetwork.com/journals/jamaneurology/fullarticle/2768084 10.1001/jamaneurol.2020.2526 PMC7341407 33165511

[55] Klunk WE (2018). Molecular imaging: what is right and what is an illusion?. Alzheimers Dement (Amst).

[56] Hanseeuw BJ, Betensky RA, Jacobs HIL, Schultz AP, Sepulcre J, Becker JA, et al. Association of amyloid and tau with cognition in preclinical alzheimer disease: a longitudinal study. JAMA Neurol. 2019;76(8):915–24. 10.1001/jamaneurol.2019.1424 Erratum in: JAMA Neurol. 2019;76(8):986. 10.1001/jamaneurol.2019.1424 PMC6547132 31157827

[57] van Berckel BNM, Ossenkoppele R, Tolboom N, Yaqub M, Foster-Dingley JC, Windhorst AD (2013). Longitudinal amyloid imaging using 11C-PiB: methodologic considerations. J Nucl Med.

[58] Barthel H, Seibyl J, Lammertsma AA, Villemagne VL, Sabri O (2020). Exploiting the full potential of β-amyloid and tau PET imaging for drug efficacy testing. J Nucl Med.

